# Tuning the Quantum Efficiency of Random Lasers - Intrinsic Stokes-Shift and Gain

**DOI:** 10.1038/srep17000

**Published:** 2015-11-23

**Authors:** Andreas Lubatsch, Regine Frank

**Affiliations:** 1 Electrical Engineering, Precision Engineering, Information Technology, Georg-Simon-Ohm University of Applied Sciences, Kesslerplatz 12, 90489 Nürnberg, Germany; 2 Physikalisches Institut, Rheinische Friedrich-Wilhelms Universität Bonn, Wegelerstr. 8, 53115 Bonn, Germany; 3 Institute of Theoretical Physics, Optics and Photonics, Eberhard-Karls-Universität Tübingen, Auf der Morgenstelle 14, 72076 Tübingen, Germany; 4 Institute of Solid State Physics, Karlsruhe Institute of Technology (KIT), Wolfgang-Gaede Strasse 1, 76131 Karlsruhe, Germany

## Abstract

We report the theoretical analysis for tuning the quantum efficiency of solid state random lasers. Vollhardt-Wölfle theory of photonic transport in disordered non-conserving and open random media, is coupled to lasing dynamics and solved positionally dependent. The interplay of non-linearity and homogeneous non-radiative frequency conversion by means of a Stokes-shift leads to a reduction of the quantum efficiency of the random laser. At the threshold a strong decrease of the spot-size in the stationary state is found due to the increase of non-radiative losses. The coherently emitted photon number per unit of modal surface is also strongly reduced. This result allows for the conclusion that Stokes-shifts are not sufficient to explain confined and extended mode regimes.

The first description of stimulated emission was given by Albert Einstein in 1916 following the consideration of Max Planck who connected a radiation field with the resonator mode. 44 years later Theodore Maiman presented the first experimental realization of a laser. Random lasers have been predicted in 1967 by Lethokov^1^,^2^. They work in principle according to the same rules as the conventional laser, only one significant difference exists: The cavity on first sight is missing^3^. Solid state random lasers consist of strongly scattering material which provides amplification on the basis of multiple scattering procedures. The amplifier consists of granular matter^4^ or a perforated amplifier, where the scattering strength is tuned with the density. The procedure is a random walk of the photon which is able to undergo interference effects if the coherence is not violated too much by inelastic scattering processes. However it does not exclude that incoherent light propagating through the sample additionally or even majorly inverts the electronic system of the material. Taking for granted that so called closed loops of traveling and interfering photons may occur, it is important to note that these effects are for sure not sufficiently strong to drive the system into lasing. They rather provide a large scale trigger which leads to stimulated emission on a well defined lasing area^5^. Hence the random laser could be a large scale single-mode laser if not local decoherence effects would actually detune it^6^. Local in this sense means that the local production of phonons at a certain position influences the energy conservation and naturally leads to a spectral broadening of the propagating photonic density. In combination with deviations in the local gain spectrum a multi-modal regime is unavoidable and experimentally observed. It is a challenge to tune the modal regime and select with respect to size, position and frequency several modes, and to deplete others^7^. A possible co-existence of extended and confined mode^8^, meaning a spatial overlap consequently means that these modes must be energetically well separated due to their gain spectrum. The question we are answering in this article is, can we tune by temperature effects, the use of a Stokes-shift during light-matter interaction, a transition between two modal regimes, can phonons be used not just to tune the laser’s frequency but also to shape the mode and how flexible is this tuning? This ansatz is not to be compared with a vibrational detuning of mesoscopic transport processes^9^. Here we focus on the electronic subsystem of the solid. The electronic transition probabilities are Stokes-detuned, which means we use a broadening of the photonic spectrum due to phonon production. Stokes-shifted photons have naturally a different mean free path and feature another absorption spectrum themselves. Such conversion increases the laser threshold of the original mode. When the number of converted photons is large, this mechanism leads to separate thresholds and so called distributed feedback lasers (DFB).

We focus our considerations here to a three dimensional thin sample that is displayed in Fig. 1. The sample is unbounded in-plane, meaning, it shall be very large compared to every other length scale in the random laser. Shown is a possible sketch of a monodisperse densly packed sample of zinc white (ZnO) spheres with a diameter of *d* = 260 *nm*. These spheres have bi-functional quality. They scatter light in the mesoscopic sense, but even more they absorb and amplify photonic intensity and act as the semi-conductor laser material. So we find two accumulation processes: First the mesoscopic accumulation which is enhanced for an increased particle density and second a retardation effect through an enhanced life-time of the electron-photon coupling in the gain region of the electronic bandstructure. This property is special for pure ZnO solid state random lasers. The density of our sample is assumed for the here presented calculations to be of 50% volume filling fraction which corresponds to experimentally accessible values^8^. Due to the high filling the scattering mean free path *l*_*s*_ of photons is comparably short and of the order of the wavelength of the scattered light. The transport velocity *v*_*t*_ hence is drastically reduced by the high number of occurring scattering events. It has to be emphasized that mesoscopic processes act as accumulation mechanism of photon density whereas the lasing frequency is dominated by the materials absorption- and emission spectrum. The semi-conductor bandstructure however may change due to intense pumping processes.

If we allow a Stokes-conversion as loss mechanism within the medium, it has to be clarified how the spot sizes of random laser modes react on this losses. The spot size is intrinsically connected to the degree of photonic correlation on the one hand side as well as to the amount of coherently emitted lasing intensity on the other hand. Our results will give insight to the relation between spot size and coherent intensity which will question the interpretation of the lasing spot as a *cavity*. This leads ultimately to the question whether a co-existing modal regime is achievable by the Stokes-tuning or whether a transition between confined and extended modes is in principle unreachable by tuning the material e.g. chemically and homogeneously in space.

## Laser Dynamics - Tuning via Stokes-Shift

**Figure 2:**
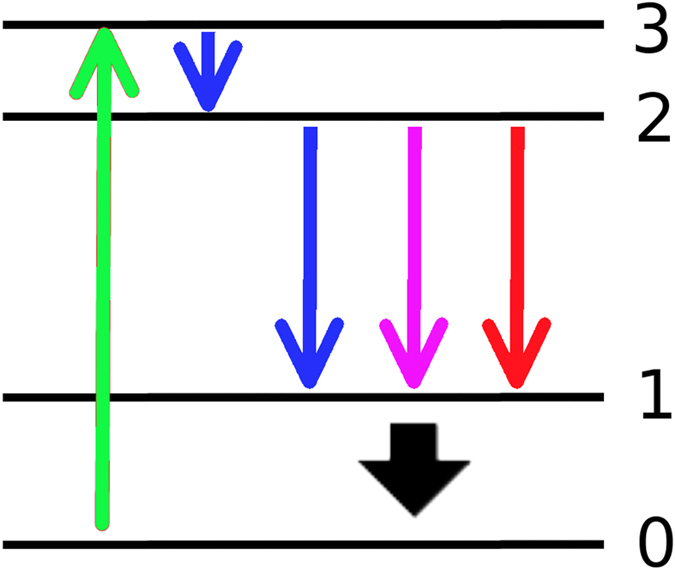
Sketch of a 4-level laser rate system. Sinuous lines represent electronic procedures due to 2-photon pumping, the excitation (green). Level 2 to 1: spontaneous emission (blue), stimulated emission (bright pink) and phonons (red).

In this work we consider ZnO nano-spheres providing the scattering and the gain-channels for random lasing action. The impinging pump laser light separates electron and atomic core within the semi-conductor lattice structure of the bulk of the pumped nano-sphere. Excitonic states are created which melt for high excitation power due to short laser pulses to an electron hole plasma. We consider here quasistationary pumping of 1.8 *MW*/*cm*^2^ to 2.4 *MW*/*cm*^2^. To describe lasing action, the electronic dynamics has to be accounted for^10^,^11^. For the atomic level laser rate system^12^ consisting of four coupled energy levels (see Fig. 2) we write


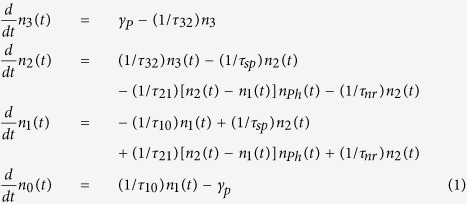


In the preceeding equations Eq. (1), *γ*_*P*_ is the external pump rate for two photon pumping, *n*_0−3_ are electronic populations of the levels respectively, *τ*_*ij*_ are the states’ lifetimes 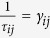, *τ*_*sp*_ represents the spontaneous decay time and *τ*_21_ is the time scale of the lasing transition. *τ*_*nr*_ is the non-radiative decay time which denotes the investigated transition in order to tune the random laser via Stokes-shift. The term [*n*_2_(*t*) − *n*_1_(*t*)]*n*_*Ph*_(*t*) marks the inversion of the occupation numbers of level 1 and 2 proportional to the number of stimulated emitted photons *n*_*Ph*_. All spatial coordinates are suppressed in Eq. (1) for the clarity of presentation, however it is noted that the photon numbers differ according to the position within the sample. The produced photon number *n*_*Ph*_ is to be read as stimulated emission per excited ZnO atom. The material is considered with a molar mass of 81,39 *g* · *mol*^−1^ and the density of 5,61 *g* · *cm*^−3^. The Stokes-shift frequency-converts a certain fraction of photons, whereas the loss (Stokes) or gain (anti-Stokes) of energy leads to a red or blue shift and so a broadening of the spontaneous emission spectra of ZnO. It has to be noted that in ZnO usually the Stokes shift is too small to generate another lasing mode. Also significant heating or cooling is expected to occur with a strong Stokes-shift. This however is not observed in ZnO but the frequency broadening is measured. Therefore the frequency-converted number of photons are simply considered as loss for the amplified mode. Consequently the non-linear feedback mechanism is working less efficiently and the threshold of the laser is increasing.

We will see in the following that not only the electronic procedures feel these losses, additionally interference effects described diagrammatically by maximally crossed diagrams (Cooperons) are reduced due to incoherent scattering. All transitions in the above described system are not independent of each other. The loss is intimately connected with the number of excited atoms and consequently with the pump intensity. This leads to the assumption that gain may compensate loss at some point, however the coherence properties of the resulting mode are fundamentally different than those of a mode in a passive, energy conserving medium.

## Self-Consistent Transport Theory of Photons

In preceeding work it has been shown that diagrammatic transport^13^,^14^ gives precise results for diffusive and localizing photons in complex random media. We constitute a diagrammatic field theory ansatz for light in a diffusive system including interferences^15^,^16^ that incorporates non-linear effects and gain. All types of light-matter interactions depend not only on the material and the passive refractive index as well as the mobility of electrons, but further on the locally impinging light intensity, the photon number. It has to be pointed out, that the refractive index of the scatterers has to be renormalized selfconsistently due to intense pumping. This is equivalent to a shift of the gain spectrum with respect to impinging intensity taking into account in this case that the threshold of a random laser is defined as the stationary state. Consequentially we treat second order non-linear response of the bulk material when the order is defined in the electromagnetic field *E*. It has been seen in the previous section, that gain processes –I*mϵ*_*s*_ lead to a retardation of coherent intensity due to a finite life-time of the electronic excitation. *ϵ*_*s*_ is the permittivity of the scatterer. Frequency conversion, in other words spectral loss or gain, leads to a change of the photon statistics respectively. The refractive index of the material is responding to these processes and especially it is responding due to spatially non-uniform or non-homogeneous procedures. These procedures are present in every system containing any boundary, meaning in any realistic setup.

Theoretically the non-linearity is established by a doubly nested self-consistency: In the following we line out the description for correlation and coherence of light in terms of the electromagnetic wave and the photon as particle.

The photon density response, the four-point correlator is derived from the Bethe-Salpeter equation of photons,


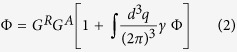


that is in the most essential form written including six independent position coordinates


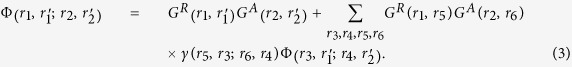


The indices mark independent positions within space. Primes denote the selfconsistency procedure of the diagram. The irreducible vertex *γ* in Eq. (2) is the heart of the formalism. It contains all possible interference effects enhanced by maximally crossed diagrams (Cooperons) which lead to a sophisticated current relaxation kernel, the memory term that renormalizes the diffusion constant *D*. We emphasize here that we assume independent monodisperse scatterers here and the Cooperon diagram is the leading order diagram under ensemble average where physically relevant system size is larger than the wavelength of light. The importance of the Cooperon has been clearly proven from our considerations of Anderson localizing systems in transmission with qualitative as well as quantitative validity^13^. The memory kernel is crucial for the random laser mode. It establishes spatial correlation and coherence whereas the temporal coherence of lasing emission is driven by the interplay between these transport processes and the non-linear response of the material. The Ward identity as such is the vital feature in photonic transport and in diagrammatic theory of two-particle propagators in general. It connects the single-particle Feynman-graph with the two-particle quantity^17^,^18^ in conserving media (*Imϵ*_*s*_ = *Imϵ*_*b*_ = 0), and in recent work^15^ it is generalized to guarantee local energy conservation, or specifically energy non-conservation for complex matter.

We write the Bethe-Salpeter equation as Boltzmann- or kinetic equation Eq. (4). The Fourier transformation and the expansion into momenta yields the exact continuity equation for the correlator Φ with spatial dependencies due to the loss channels at the boundaries of the finite system and additionally the current density relation.


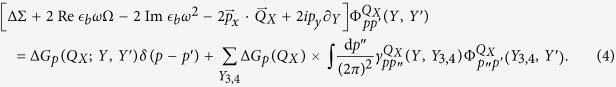


Δ*G* = *G*^*R*^ − *G*^*A*^. *p*, *p*′ and *p*″ are momenta. The scatterer’s geometric properties are represented within the self-consistent complex valued scattering matrices *T* of the Schwinger-Dyson equation *G* = *G*_0_ + *G*_0_*TG* which leads to the solution for the Green’s function *G*^*R*^ and *G*^*A*^ of the electromagnetic field, the light wave. The ZnO scatterer’s initial permittivity is given by *Reϵ*_*s*_ = 4.0164, the imaginary part *Imϵ*_*s*_, the microscopic gain, is computed self-consistently yielding gain saturation. The photon density emitted from the amplifying Mie particles is derived by means of coupling to the rate equation system (see previous section). It is self-consistently connected the dielectric function *ϵ* = *ϵ*_*L*_ + *ϵ*_*NL*_. Finally we arrive at nonlinear feedback in both, electromagnetic wave transport and photon intensity transport for scalar waves. The scalar approach is especially suitable to model absolutely randomized particle systems. Further the Mie character develops with reducing particle size into a Rayleigh scatterer and strong non-isotropies which might influence the vector-character are consequentially not given. Only in setups of pronounced Mie type scatterers as well as order or quasi-order we expect the vector-character of light to become important. Random lasing in such setups has not been investigated theoretically as well as experimentally so far.

Within grand canonical (open) ensembles of random lasers the entropy is increased by photonic intensity transport processes. Nevertheless transport in the meaning at hand is based on the time reversal symmetry of the single particle Green’s function 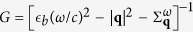 describing the propagation of the electromagnetic wave. This time reversal symmetry is diagrammatically not broken. We describe the laser dynamics within a laser rate equation system that is suitable for non-linear processes or quantum cascades^12^. The advantage is obvious since nonradiative decay processes within that system act directly on the electronic subsystem, the particle, and so enter directly the non-linear complex refractive index and the self-energy Σ of the single independent Mie scatterer, modelled as the complex scattering or **T** matrix. For clarity it shall be pointed out that the complex refractive index acts equally absorbing or emitting under time reversibility. Microcanonically the time evolution is flipped, however the system evolves grand-canonically *open*^14^.

The described procedure of modeling disorder and dissipation guarantees the completeness of the ‘ab initio’ description of the propagating light intensity by the four-point correlator Φ = *A*Φ_*ϵϵ*_ + *B*Φ_*Jϵ*_ here given in terms of the momenta. Φ_*ϵϵ*_ equals the energy density and Φ_*Jϵ*_ equals the energy current, *A* and *B* are pre-factor terms derived in^16^. The framework yields all specific transport characteristics, e.g. the scattering mean free path *l*_*s*_ and includes all interference effects. The mode is described efficiently by the determination of the correlation length *ξ* with respect to various loss channels. This length *ξ* in non-linear systems marks a decay of the intensity to 1/*e*. It is of a qualitative different importance than the localization length in the Anderson sense^14^, because the diffusion constant is *D*≠0 in complex media. In other words, the state in this case has a finite lifetime compared to the immanent infinite lifetime of an Anderson localized state in a passive system. The Bethe-Salpeter equation is solved in a sophisticated regime of real space and momentum and the description for the energy density Φ_*ϵϵ*_(*Q*, Ω) is derived which is computed regarding energy conservation


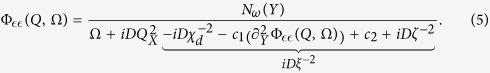


The numerator *N*_*ω*_ is the local density of photonic modes LDOS renormalized due to amplification and absorption of the electromagnetic wave. *Q* equals the center of mass momentum of the propagator denoted in Wigner coordinates, Ω is the center of mass frequency and *D* is the self-consistently derived diffusion constant. *c*_1_, *c*_2_ are coefficients having a non-trivial form of only numerical relevance. Following the formal analytical structure of ref. 16 up to the result of Eq. (28) under the additional assumption of boundaries as well as the coupling to laser rate equations (Eq. (1)) leads actually to some more sophisticated term. This result has to be reformed algebraically until it fits in it’s structure a formal diffusion pole again. The above mentioned form of the energy density Φ_*ϵϵ*_ (Eq. (5)) emerges. A divergence, that is marked by critical scales in the unlimited system of^16^, is here instead replaced by the phase transition towards lasing. Consequentially modes derived as the characterising result here equal not only coherent transport of photons but they rather equal lasing modes caused by the inverted electronic sub-system in quasi-equilibrium, the stationary state.

The structure of the Bethe-Salpeter equation and the diffusion pole will be discussed in the following. The excitation process is uniform in space. Interferences gain weight on long paths in-plane of the large scaled random laser sample. The physics of maximally crossed diagrams therefore significantly dominates the coherence properties: Dissipation and losses due to spontaneous emission and non-radiative decay are in principle homogeneous, however they depend of course very well on the impinging energy density and the resulting non-linear response. As consequence these properties change with the position relative to the samples boundaries if the latter are lossy. All channels are represented within the pole of Eq. (5) resulting in separate dissipative length scales *ζ* due to homogeneous losses, and *χ*_*d*_ due to gain and absorption that go along with photonic transport and the open or strongly absorbing boundaries. All dissipation processes enter the mass term of the diffusion equation:


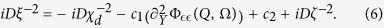


By solving of the non-classical diffusion equation Eq. (6) the coefficients *c*_1_ and *c*_2_ are selfconsistently derived. Non-classical is defined as to consider light, as explained above, diagrammatically not just as a wave but in addition as particle (photon) current. Finally, we derive the spatial distribution of energy density:


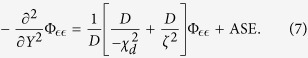


The nonlinear self-consistent microscopic random laser gain *γ*_21_*n*_2_ incorporates the influences of both length scales *χ*_*d*_ and *ζ*,


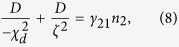


and therefore represents the physical properties of the random laser samples within the absorptive boundaries. *γ*_21_ is the transition rate of stimulated emission and *n*_2_ equals the selfconsistent occupation of the upper laser level. The abbreviation ASE on the right of Eq. (7) represents all transport terms yielding amplified spontaneous emission.

## Results and Discussion

In the previous sections we developed a model for transport in strongly scattering, dense particle agglomerates. Non-linear gain and gain saturation is included in the model Fig. 2 by the coupling to a rate equation system, describing the full lasing dynamics Eq. (1).

**Figure 3:**
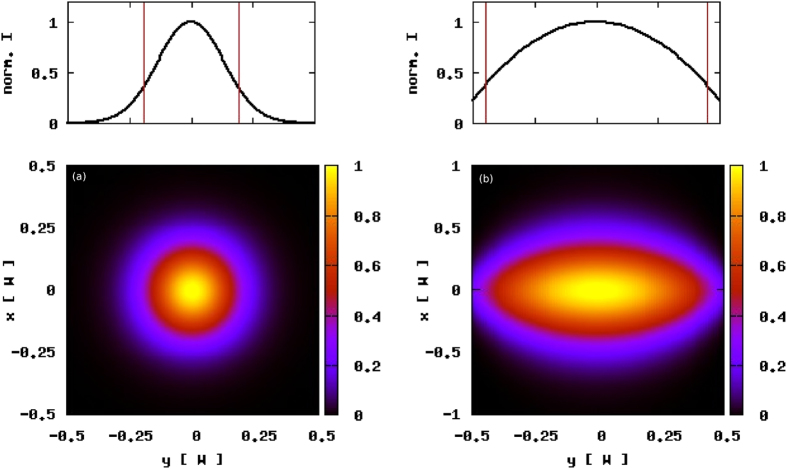
Computed coherent lasing intensity distribution (color bar). The ZnO scatterers, diameter *d* = 260 *nm*, filling 50%, are embedded in a lossy substrate i.e. GaAs or SiO. The dimensions are *W*_*x*_ = 20.0 *μm*, *W*_*y*_ >> 40.0 *μm*, *W*_*z*_ = 4.0 *μm*. Results are shown for homogeneous 2-photon pumping *λ* = 355 nm (bandedge of ZnO bulk). (a) Confined mode. Emission energy is 3.23 *eV*, the transport mean free path *l*_*s*_ = 499.2 nm. Shown is the result onsight on the samples section of 20.0 *μm* × 20.0 *μm*, the mode features no absorption at the samples edges. However the underlying substrate is absorbing. (b) Extended mode. Sample section 20.0 *μm* × 40.0 *μm*. The profiles above the color coded plots show the normalized coherent intensity *I* of each mode. The difference due to lossy boundaries in (b) is evident by comparing the decay to 1/*e* (vertical lines in (a) and (b)). The phonon-production in this calculation is *γ*_*NR*_ = 0.

In Fig. 3 we display the onsight on a lasing sample derived by calculations for co-existing random lasing modes of ZnO powder on GaAs substrate. The co-existence is explained by a sharp spectral separation of both mode types. Extended modes may only be developed due to absorption at the samples boundaries as it has been shown in^5^. In these calculations the non-radiative decay *γ*_*nr*_ is assumed to be 0, so the sample features only losses at the boundaries towards the substrate and out of plane in *z*–direction. Lasing principle occurs in all D = 3 dimensions but lasing intensity escapes the sample only in the *z*–direction. Under the additional premise that the observed lasing frequency is not absorbed at the samples boundaries in-plane (*x* − *y* − plane), which means the sample size is infinite compared to the mean free path *l*_*s*_, confined modes arise Fig. 3(a). Our result is experimentally confirmed in samples of ZnO on SiO and GaAs^8^.

**Figure 4:**
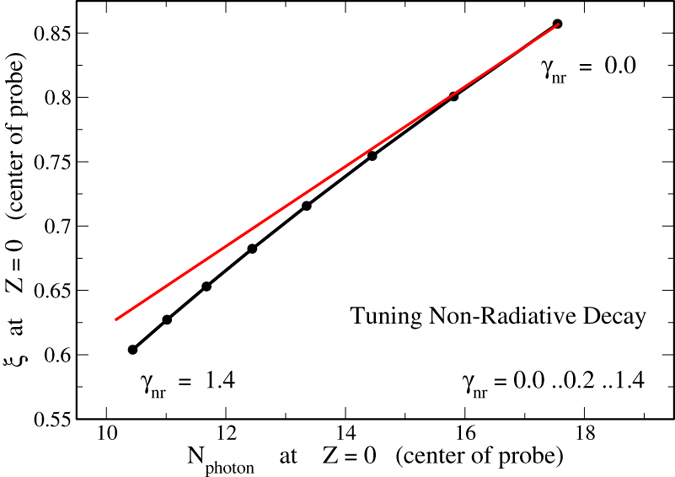
The computed correlation length *ξ*, the mode diameter of a bulk mode at the surface, as a function of the emitted photon number in the center of a confined mode which is tuned by non-radiative decay. The decay rate *γ*_*NR*_ varys between 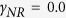 and 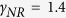 in units of the spontaneous emission rate. The non-linear behavior (indicated by deviation from a linear behavior given as red line) is clearly enhanced dependent to strong losses and the mode diameter is reduced about 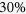. It is evident that the mode is continuously decreased in the confined regime.

We are tuning now the phonon-production rate in order to investigate thermal loss as an adjustment process for random laser modes. This is equivalent with a microscopic detuning of the electronic subsystem, in other words a modified quantum efficiency. Phonon production as a homogeneous rated process in the whole system in first instance reduces of course spontaneous emission and therefore rises the laser threshold as such. As a second process that is subtle but even more important, it reduces coherent scattering and interference effects. Long range interferences, represented in the Cooperon diagram, however, trigger stimulated processes on large scales. If they are reduced, not only the amount of coherently emitted intensity is smaller, also the modes diameters generally are small Fig. 4. The sample is changing it’s quality from the laser, which features a phase transition at the threshold, towards a so called super-radiating system that has the functionality of a large area light-emitting diode (LED). In Eq. (7) the last term responsible for amplified spontaneous emission (ASE) gradually is increased. The numerical analysis at the laser threshold can be found in Fig. 3(a) and 4. For the confined modes it is found, that they behave non-linear when the phonon-production is modified. In Fig. 4 the diameter of the mode with varying *γ*_*NR*_ is displayed. *γ*_*NR*_ is running over 0.0..1.4 in units of the spontaneous transition rate. The dots on the curve mark equidistant steps of 0.2. It is found in the deviation from the red line that the decrease of the diameter is non-linearly behaving with the loss. Also the photon emission rate shows a non-linearity but with an opposite impact. The distance between the dots for increasing *γ*_*NR*_ is decreasing. This can be understood in the picture of the Cooperon as the stimulating process for lasing radiation as explained above.

Both modal regimes are not just simply varying in their diameter, they are in our theory rather different from the fundamental point of view. It can be excluded that non-radiative tuning will cause another modal regime like an extended mode covering the whole sample as displayed in Fig. 3(b). This transition is not to be explained as an intrinsic Stokes-shift. It is further noted that the extended mode, which is connected by loss to the surrounding substrate suffers through additional non-radiative losses in intensity. The amount of coherently emitted intensity is reduced. However their mode diameters are almost insensitive to that loss type because they are pinned to the bondaries.

## Summary and Conclusion

We have shown in this work co-existing extended and confined random laser modes. Extended modes occur according to our results definitely due to boundary absorption. Tuning the quantum efficiency of large samples of random lasers by means of non-radiative decay leads to a modulation of the lasing spot size of the modes in the confined regime. However, in our framework we derive a non-linear dependency and a decrease of the spot diameters with an increasing phononic action. The transition from confined to extended modes, so the drastic increase of the mode diameter, is certainly not reachable by temperature or phonon production. Additionally we have found in our theoretical analysis evidence that the Cooperon contribution is reduced by absorption and supported by gain procedures even though the Anderson transition in it’s original sense is not given in open random media. However it will be extremely interesting to understand these fundamental procedures and to find out numerically in detail, how the interplay of diffusion and localization procedures is responding to gain. Up to our knowledge such a study has not been performed yet.

## Additional Information

**How to cite this article**: Lubatsch, A. and Frank, R. Tuning the Quantum Efficiency of Random Lasers - Intrinsic Stokes-Shift and Gain. *Sci. Rep.* **5**, 17000; doi: 10.1038/srep17000 (2015).

## Figures and Tables

**Figure 1:**
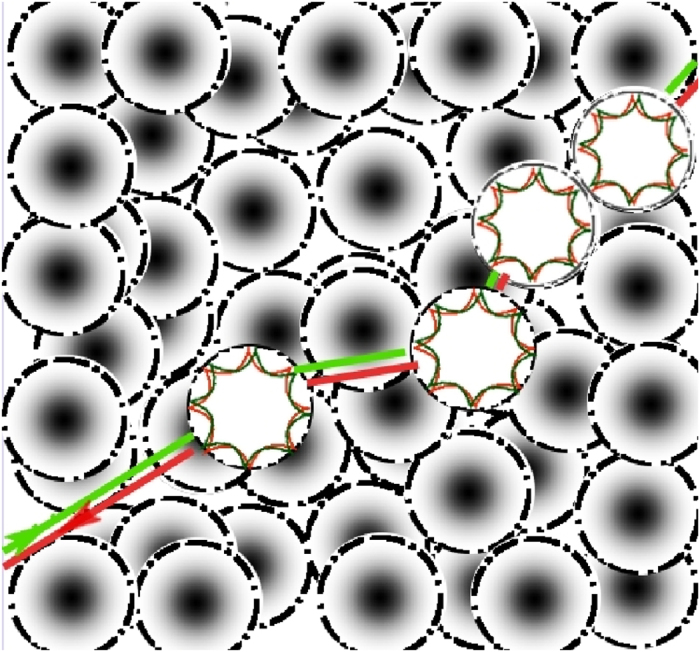
Sketch of experimentally relevant sample. Displayed is a powder of monodisperse ZnO grains, diameter of the grains is *d* = 260 *nm*. The volume filling of the sample is assumed to be about 50%. The sample shall be thin in the direction of the incident pump beam but infinite in-plane. The red and green paths mark the propagation direction of scattered photons (red) and their time-reversal symmetric processes (green). The full correlation includes all interferences in the theory. Within the spheres photons experience amplification through extended light-matter bound states as well Mie resonances. They can be seen as whispering gallery modes propagating inside the sphere and being reflected at the surface determined by the refractive index contrast, which enhance forward-scattering^19^.
